# Hypomyelination Leukodystrophy 16 (HLD16)-Associated Mutation p.Asp252Asn of TMEM106B Blunts Cell Morphological Differentiation

**DOI:** 10.3390/cimb46080478

**Published:** 2024-07-27

**Authors:** Sui Sawaguchi, Miki Ishida, Yuki Miyamoto, Junji Yamauchi

**Affiliations:** 1 Laboratory of Molecular Neurology, Tokyo University of Pharmacy and Life Sciences, Tokyo 192-0392, Japanmiyamoto-y@ncchd.go.jp (Y.M.); 2 Laboratory of Molecular Pharmacology, National Research Institute for Child Health and Development, Tokyo 157-8535, Japan; 3 Diabetic Neuropathy Project, Tokyo Metropolitan Institute of Medical Science, Tokyo 156-8506, Japan

**Keywords:** TMEM106B, oligodendrocyte, differentiation, hesperetin, mTOR

## Abstract

Transmembrane protein 106B (TMEM106B), which is a type II transmembrane protein, is believed to be involved in intracellular dynamics and morphogenesis in the lysosome. TMEM106B is known to be a risk factor for frontotemporal lobar degeneration and has been recently identified as the receptor needed for the entry of SARS-CoV-2, independently of angiotensin-converting enzyme 2 (ACE2). A missense mutation, p.Asp252Asn, of TMEM106B is associated with hypomyelinating leukodystrophy 16 (HLD16), which is an oligodendroglial cell-related white matter disorder causing thin myelin sheaths or myelin deficiency in the central nervous system (CNS). However, it remains to be elucidated how the mutated TMEM106B affects oligodendroglial cells. Here, we show that the TMEM106B mutant protein fails to exhibit lysosome distribution in the FBD-102b cell line, an oligodendroglial precursor cell line undergoing differentiation. In contrast, wild-type TMEM106B was indeed localized in the lysosome. Cells harboring wild-type TMEM106B differentiated into ones with widespread membranes, whereas cells harboring mutated TMEM106B failed to differentiate. It is of note that the output of signaling through the lysosome-resident mechanistic target of rapamycin (mTOR) was greatly decreased in cells harboring mutated TMEM106B. Furthermore, treatment with hesperetin, a citrus flavonoid known as an activator of mTOR signaling, restored the molecular and cellular phenotypes induced by the TMEM106B mutant protein. These findings suggest the potential pathological mechanisms underlying HLD16 and their amelioration.

## 1. Introduction

Transmembrane protein 106B (TMEM106B) is a type II transmembrane protein [1] whose membrane protein family generally plays a key role not only in sustaining various organelle functions but also in cell morphogenesis and, in turn, tissue development [1,2,3,4]. TMEM106B is specifically localized in the late endosome and lysosome membranes [1,2].

Detailed studies using mice illustrate that TMEM106B is expressed primarily in oligodendroglial precursor cells and early maturating oligodendroglial cells in the central nervous system (CNS) [2]. The expression profiles of TMEM106B are preserved in humans and TMEM106B exhibits high expression levels in oligodendroglial lineage cells [2]. Interestingly, the overexpression of TMEM106B in mouse oligodendroglial cells enlarges the lysosomal size, leading to dysfunction in the endosomal and lysosomal system and abnormal cell morphologies [3,4], underscoring the critical relationship between TMEM106B and oligodendroglial cell morphogenesis.

On the other hand, in rat hippocampal and cortical neurons, the decreased expression of the TMEM106B protein results in enhanced retrograde lysosomal trafficking in neuronal dendrites, reducing their branching [5]. In this context, the knockdown of microtubule-associated protein 6 (MAP6) restores the balance of anterograde and retrograde lysosomal trafficking, preventing dendrite loss [5]. It is therefore thought that TMEM106B regulates lysosome morphologies, cell morphologies, and putative cellular functions in both oligodendroglial and neuronal cells [5,6].

Hypomyelinating leukodystrophies (HLDs) are a group of hereditary neuropathies involving thin and aberrant oligodendroglial cell plasma membrane-derived myelin membranes in the CNS [7,8,9,10,11,12]. Oligodendroglial cells are a type of glial cell in the CNS and are responsible for the formation of myelin sheaths, which are essential in controlling saltatory conduction and protecting nerve axons [13,14,15,16]. HLDs are rare, occurring in 1 in 250,000 to 500,000 people. Typical clinical features include impaired motor development, progressive limb spasticity, nystagmus, and a gradual decline in cognitive function [7,8,9,10,11,12]. The responsible genes have been identified following advances in nucleotide sequencing technologies, including next-generation sequencing (NGS) techniques [9,10]. TMEM106B is the gene product identified by such NGS techniques as being responsible for HLD type 16 (HLD16) [2]. The missense mutation p.Asp252Asn (an Asp252-to-Asn mutation) is associated with HLD16 [2]. Brain magnetic resonance imaging (MRI) demonstrates that HLD16 has many typical HLD symptoms, with thin myelin sheaths or some brain regions exhibiting complete myelin deficiency. In addition, many cases display common clinical symptoms, including nystagmus and motor impairment, as well as characteristic symptoms such as hypotonia [2,17,18,19]. There are no known treatments for HLD16, including thin myelin sheath-related dementia associated with TMEM106B [2,17,18,19,20].

Herein, we report that the HLD16-associated TMEM106B mutant protein fails to exhibit lysosome distribution in the FBD-102b cell line, a mouse oligodendroglial cell line involved in the formation of myelin-like widespread membranes [21,22,23,24]. In contrast, the wild-type TMEM106B was indeed localized in the lysosome. While cells harboring the wild-type TMEM106B exhibited morphologically differentiated phenotypes, those harboring TMEM106B p.Asp252Asn failed to differentiate. Decreased levels of phosphorylation of lysosome-related mechanistic target of rapamycin (mTOR) signaling output molecules, such as the ribosomal S6 and translational 4E-BP1 proteins, were observed in cells harboring mutated TMEM106B. Hesperetin, a citrus flavonoid, is known for its role as an mTOR signaling activator [25,26] and for its neuronal and glial protective effects [27,28]. Hesperetin resulted in the retention of these phenotypic defects. These results suggest that the HLD16-associated mutation of TMEM106B may contribute to blunted morphological differentiation through the decreased phosphorylation of the ribosomal S6 protein and 4E-BP1.

## 2. Materials and Methods

### 2.1. Antibodies, Plasmids, and siRNAs

The key antibodies and plasmids are listed in Table 1.

### 2.2. Cell Culture, Differentiation, and Image Capture

The FBD-102b cell line is a mouse oligodendroglial precursor cell line (Riken, Saitama, Japan). Cells were cultured on Nunc cell culture dishes (Thermo Fisher Scientific, Waltham, MA, USA) in Dulbecco’s modified Eagle medium (DMEM)/F-12 medium (Nacalai Tesque, Kyoto, Japan; Fujifilm, Tokyo, Japan)/10% heat-inactivated fetal bovine serum (FBS)/Pen-Strep mixture (Thermo Fisher Scientific) in 5% CO_2_ at 37 °C.

In order to induce differentiation, polylysine (Nacalai Tesque)-coated cell culture dishes were cultured in medium with 1% fetal bovine serum for several days in 5% CO_2_ at 37 °C in the presence or absence of hesperetin. Cells with secondary branches from primary ones or with myelin membrane-like widespread membranes (cells large enough to contain a circle with a diameter of more than 50 μm) were considered to represent differentiated phenotypes [23,24].

The number of attached cells incorporating trypan blue (Nacalai Tesque) was estimated to be less than 5% under these conditions [23,24]. Cell morphologies were captured and preserved using microscopic systems equipped with i-NTER LENS (Micronet, Saitama, Japan). The images in the figures are representative of multiple images and were analyzed with the Image J software ver. 1.54j (https://imagej.nih.gov/, accessed on 1 March 2024).

### 2.3. Transient and Stable Transfection

In accordance with the manufacturer’s instructions, the respective plasmids were transfected using the ScreenFect A or ScreenFect A Plus transfection kit (Fujifilm). The medium was replaced 4 h after transfection and generally used for more than 48 h after transfection for experiments.

Stable clones were collected in the presence of 1 mg/mL of G418 (Nacalai Tesque) in accordance with the manufacturer’s instructions. Under these conditions, the number of attached cells incorporating trypan blue was estimated to be less than 5% in each experiment at 48 h after transfection.

### 2.4. Fluorescence Images

Cells on coverslips were fixed with 4% paraformaldehyde (Nacalai Tesque) or 100% cold methanol (Nacalai Tesque) and blocked with Blocking One (Nacalai Tesque). Slides were incubated with primary specific antibodies preloaded with fluorescent dye-conjugated secondary antibodies. The coverslips were mounted using the Vectashield mixture (Vector Laboratories, Burlingame, CA, USA). Fluorescent images were collected and merged with a microscopic system, FV3000 or FV4000, equipped with a laser scanning Fluoview apparatus (both from Olympus, Tokyo, Japan), or the BZ-X700 microscopic system equipped with a fluorescence apparatus (Keyence, Tokyo, Japan). The images in the figures are representative of multiple images and were analyzed using the Image J software. Merged percentages are statistically depicted using merged image pixel values (yellow fluorescence) relative to the image values showing TMEM106B (green fluorescence).

### 2.5. Cell Lysis and Polyacrylamide Gel Electrophoresis

Lysis buffer (50 mM HEPES-NaOH, pH 7.5, 150 mM NaCl, 3 mM MgCl_2_, 1 mM dithiothreitol, 1 mM phenylmethane sulfonylfluoride, 1 μg/mL leupeptin, 1 mM EDTA, 1 mM Na_3_VO_4_, 10 mM NaF, and 0.5% NP-40) was used for lysing [23,24]. Cell lysates were denatured in pre-made sample buffers (Nacalai Tesque or Fujifilm). The denatured samples were separated on pre-made sodium dodecyl sulfate–polyacrylamide gel (Nacalai Tesque or Fujifilm).

### 2.6. Immunoblotting

The electrophoretically separated proteins were transferred to polyvinylidene fluoride membranes (Fujifilm). The membranes were blocked with Blocking One. They were immunoblotted using primary specific antibodies and in turn using peroxidase enzyme-conjugated secondary antibodies. The immunoreactive bands in the membranes were captured and preserved using the CanoScan LiDE 400 system (Canon, Tokyo, Japan). Multiple sets of experiments were conducted in the immunoblotting studies, and the quantification of the immunoreactive bands, using another sample’s immunoreactive band as 100%, was performed with the Image J software.

### 2.7. Immunoprecipitation of Intact Intracellular Components

Cells were extracted in an isotonic extraction buffer (50 mM HEPES-NaOH, pH 7.5, 125 mM NaCl, 3 mM MgCl_2_, 1 mM phenylethane sulfonylfluoride, 1 μg/mL leupeptin, 1 mM EDTA, 1 mM Na_3_VO_4_, and 10 mM NaF) with short-term sonication. Cell extracts were used for immunoprecipitation with the respective antibodies against intact intracellular components [29,30]. The denatured, immunoprecipitated samples containing intracellular components were separated on pre-made sodium dodecyl sulfate–polyacrylamide gel for subsequent immunoblots using specific antibodies.

### 2.8. Statistical Analysis

Values are means ± standard deviation (SD) from separate experiments. Intergroup comparisons were performed using the unpaired *t*-test with Student’s or Welch’s correction in the Excel software (Microsoft, Redmond, WA, USA).

Differences were considered significant at less than 0.05. For all analyses, the investigator was blinded to the sample conditions.

### 2.9. Ethics Statement

In accordance with a protocol approved by the Tokyo University of Pharmacy and Life Sciences Gene and Animal Care Committee (Approval Nos. LS28-20 and LSR3-011), techniques using genetically modified cells and related techniques were performed.

## 3. Results

### 3.1. Mutated TMEM106B Fails to Be Localized around the Lysosome, Whereas Wild-Type TMEM106B Is Localized around the Lysosome

**Figure 1:**
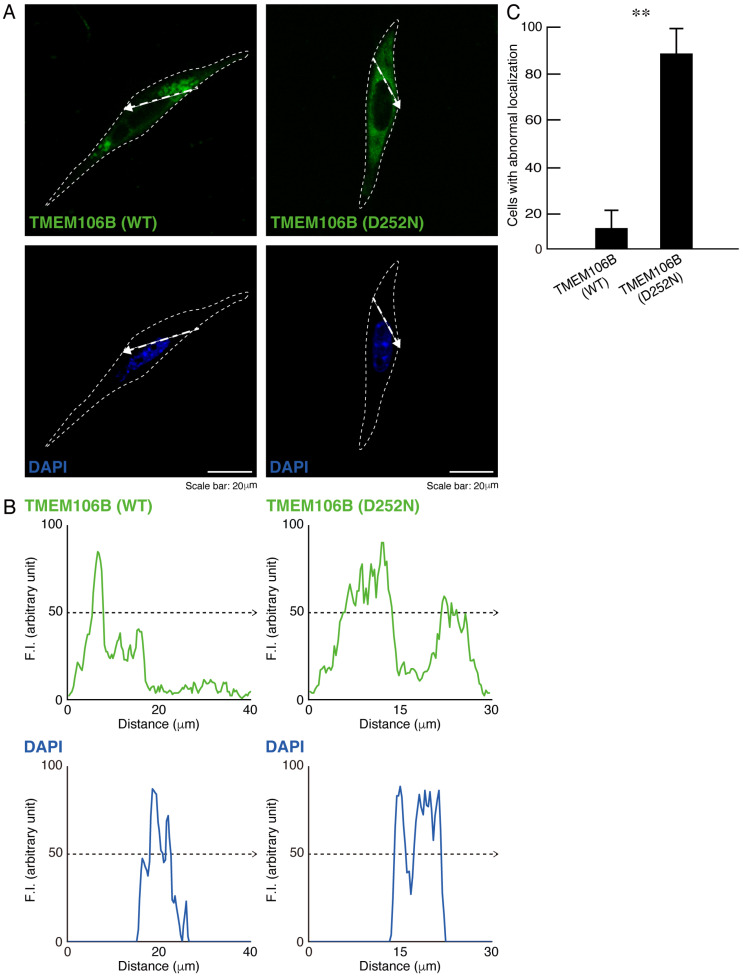
TMEM106B protein with the HLD16-associated D252N mutation is widely distributed throughout the cytoplasmic regions. (**A**) FBD-102b cells (surrounded by white dotted lines) were transfected with the plasmid encoding wild-type (WT) TMEM106B tagged with EGFP at its C-terminus or EGFP-tagged TMEM106B with the D252N mutation. Transfected cells were stained with DAPI for nuclear staining. Scan plots were created along the white dotted lines in the direction of the arrows in the images. (**B**) Graphs showing fluorescence intensities (F.I., arbitrary units) along the white dotted lines in the direction of the arrows are presented at the bottom of the representative fluorescence images. (**C**) Cells with abnormal, widely distributed structures were counted and statistically depicted (** *p* < 0.01; *n* = 10 fields).

To explore whether mutated TMEM106B changes its intracellular localization, we transfected the plasmid encoding GFP-tagged mutated *TMEM106B* in FBD-102b cells. Transfected wild-type TMEM106B exhibited lysosome organelle-like punctate structures in cells [2,3,4], whereas mutated TMEM106B failed to be distributed primarily in punctate structures (Figure 1A,B). Overall, 90% of the cells expressing mutated TMEM106B exhibited abnormal localization (Figure 1C).

**Figure 2:**
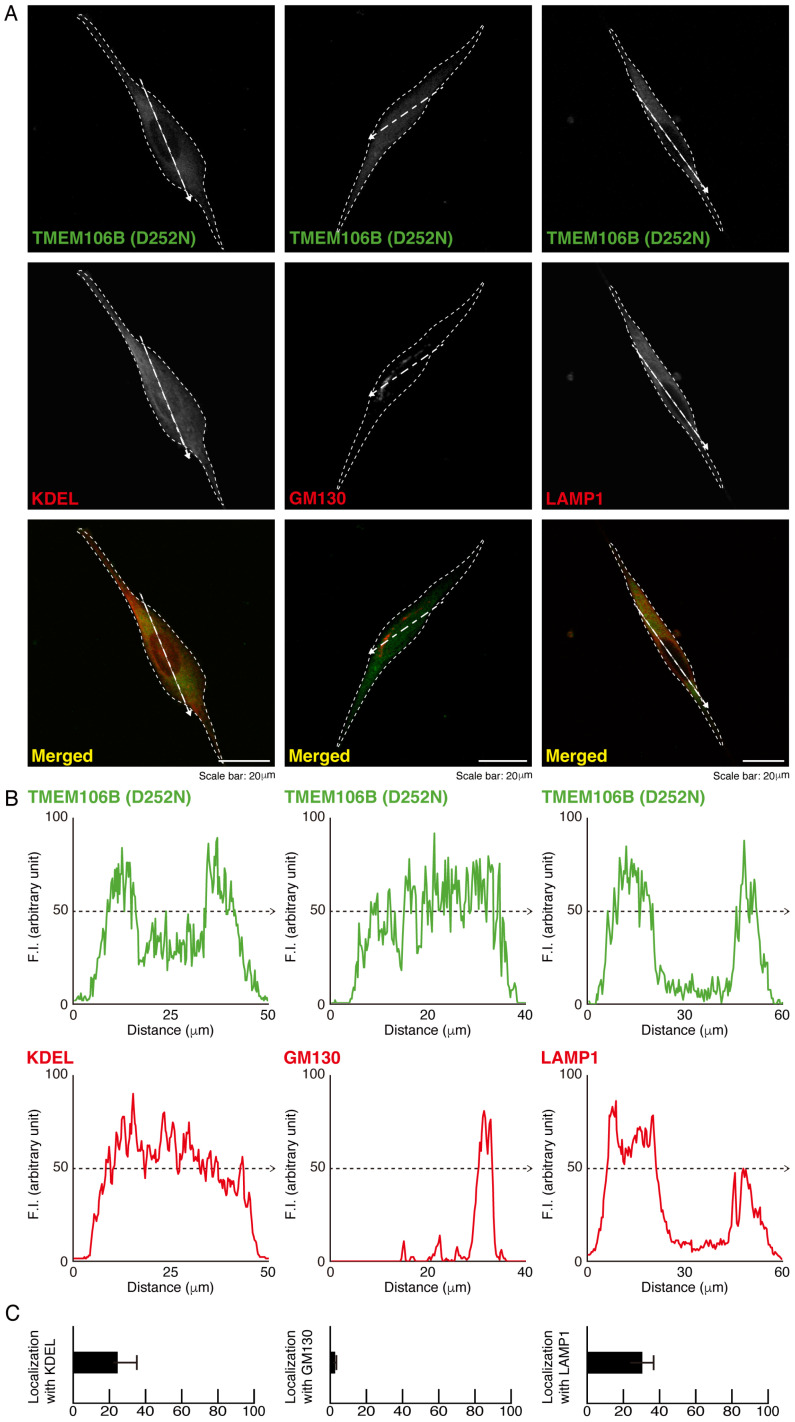
Mutated TMEM106B is present in the lysosome. (**A**) Cells (surrounded by white dotted lines) were transfected with the plasmid encoding mutated TMEM106B (D252N). Transfected cells were stained with the respective antibodies against ER-specific KDEL, Golgi body-specific GM130, and lysosome-resident LAMP1. Scan plots were created along the white dotted lines in the direction of the arrows in the images. (**B**) Graphs showing fluorescence intensities (F.I., arbitrary units) along the white dotted lines in the direction of the arrows are presented at the bottom of the representative fluorescence images. (**C**) The respective merged percentages are depicted in bar graphs (*n* = 3 fields).

Thus, we investigated whether mutated TMEM106B was preferentially localized in the lysosome. We stained cells transfected with mutated TMEM106B with the respective organelle antibodies. Mutated TMEM106B did not primarily colocalize with antibodies against endoplasmic reticulum (ER)-specific Lys-Asp-Asn-Leu (KDEL) antigen, 130 kDa Golgi body protein GM130 antigen, and lysosomal-associated membrane protein 1 (LAMP1) antigen (Figure 2A–C), suggesting that mutated TMEM106B is mostly unable to localize to the lysosome.

To investigate whether mutated TMEM106B was present in other small vesicular intracellular components, we prepared extracts from cells transfected with the plasmid encoding EGFP-tagged *TMEM106B* or the mutant protein in an isotonic solution. Immunoprecipitation data using antibodies against Arf6 as the vesicular marker protein around the plasma membrane, the early endosome marker Rab5, and the late endosome marker Rab7 [29,30,31,32] illustrated that the mutant protein, but not the wild-type version, was present in a Rab7-positive intracellular component (Figure S1). The expression levels of transfected proteins and marker proteins were comparable in cells expressing mutated and wild-type TMEM106B.

### 3.2. Cells Harboring Mutated TMEM106B Fail to Exhibit Differentiated Phenotypes, Whereas Cells Harboring Wild-Type TMEM106B Can Exhibit Them

**Figure 3:**
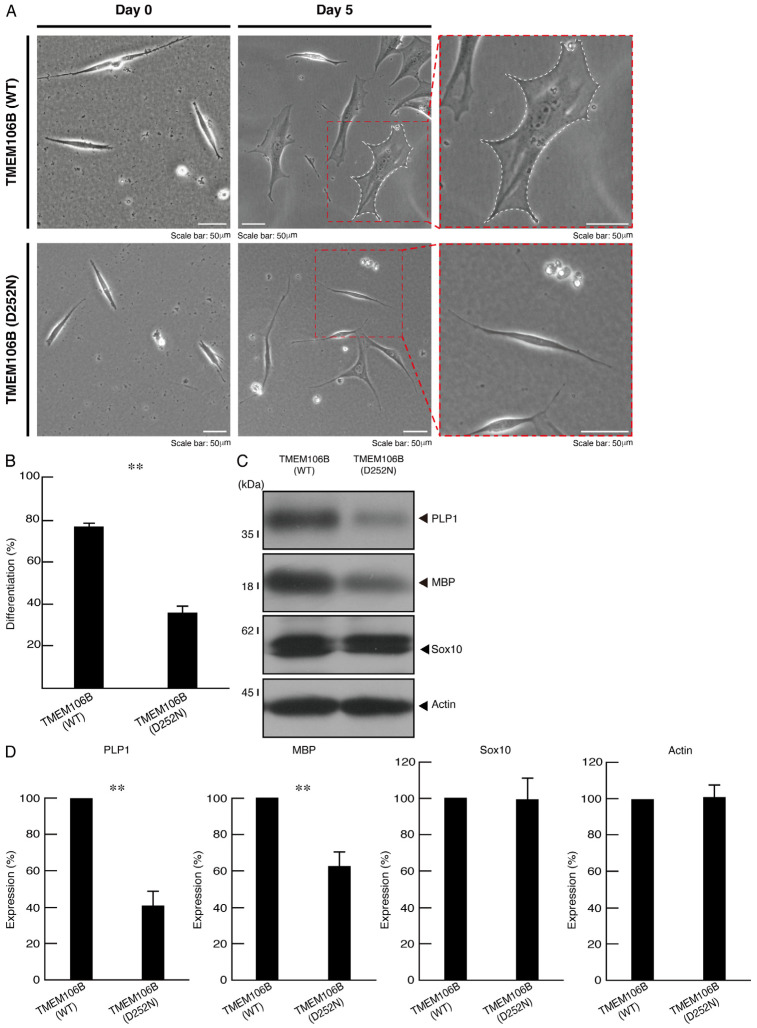
Cells harboring mutated TMEM106B show decreased cell differentiation abilities. (**A**) Cells harboring wild-type (WT) or mutated (D252N) TMEM106B were allowed to differentiate for 0 or 5 days. Cells surrounded with dotted red lines in the middle panels are magnified in the right panels. The cell surrounded by a white dotted line is a typically differentiated one with widespread membranes. (**B**) Differentiated cells are statistically depicted (** *p* < 0.01; *n* = 10 fields). (**C**) The lysates of cells at 5 days following the induction of differentiation were immunoblotted with the respective antibodies against differentiation markers PLP1 and MBP, cell lineage marker Sox10, and internal control actin. (**D**) Quantification of immunoreactive bands, using control immunoreactive bands as 100%, is depicted in the respective graphs of PLP1, MBP, Sox10, and actin (** *p* < 0.01; *n* = 3 blots).

Since mutated TMEM106B is unable to localize to lysosomes, we examined whether it also affected the morphological differentiation in FBD-102b cells. Following the induction of differentiation, cells harboring mutated TMEM106B failed to exhibit differentiating phenotypes with widespread membranes; in contrast, cells harboring wild-type TMEM106B achieved differentiation (Figure 3A,B). These phenotypes were consistent with the decreased expression levels of oligodendrocyte differentiation/myelination marker proteins proteolipid protein 1 (PLP1) and myelin basic protein (MBP) in cells harboring mutated TMEM106B (Figure 3C,D). Oligodendrocyte lineage Sox10 and internal control actin proteins were comparable in cells harboring mutated and wild-type TMEM106B. To confirm the effect of mutated TMEM106B on the differentiating morphologies, we stained the cells with an antibody against MBP. Mutated TMEM106B resulted in decreased differentiation phenotypes (Figure S2A,B).

**Figure 4:**
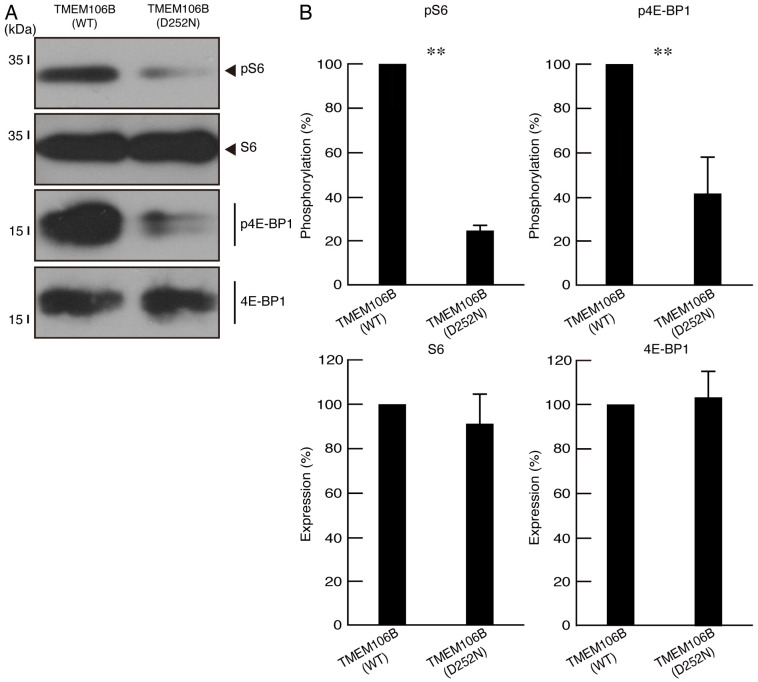
Cells harboring mutated TMEM106B show decreased phosphorylation levels of ribosomal S6 and translational 4E-BP1 proteins. (**A**) The lysates of cells at 5 days following the induction of differentiation were immunoblotted with the respective antibodies against phosphorylated ribosomal S6 and translational 4E-BP1 proteins (pS6 and p4E-BP1). Total ribosomal S6 and translational 4E-BP1 protein (S6 and 4E-BP1) bands are also presented. (**B**) Quantification of immunoreactive bands, using control immunoreactive bands as 100%, is depicted in the respective graphs of pS6, S6, p4E-BP1, and 4E-BP1 (** *p* < 0.01; *n* = 3 blots).

Next, we checked the phosphorylation levels of the ribosomal S6 and translational 4E-BP1 proteins as the output molecules of mTOR signaling, which is essential for oligodendrocyte differentiation/myelination [15,16]. As expected, their phosphorylation levels were greatly decreased in cells harboring mutated TMEM106B, whereas the protein expression levels of ribosomal S6 and translational 4E-BP1 were comparable in cells harboring mutated and wild-type TMEM106B (Figure 4A,B), indicating that mutated TMEM106B has the ability to decrease morphological differentiation.

### 3.3. Hesperetin Recovers Phenotypes in Cells Harboring Mutated TMEM106B

**Figure 5:**
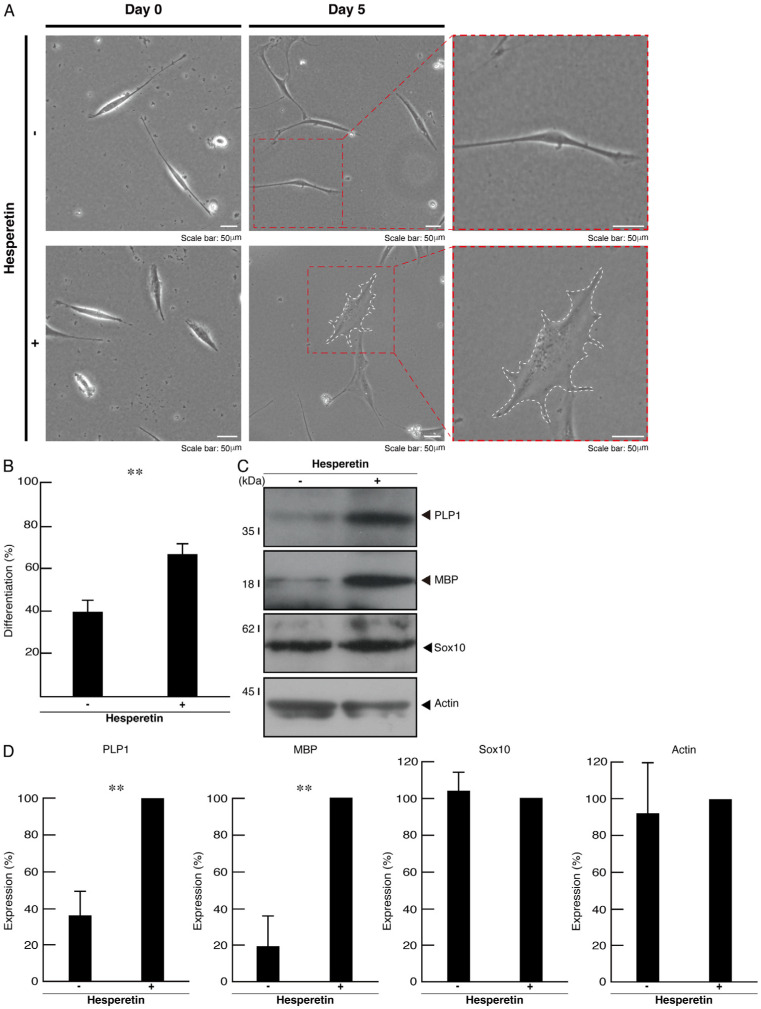
Hesperetin recovers phenotypes of cells harboring mutated TMEM106B. (**A**) Cells harboring mutated TMEM106B were allowed to differentiate for 0 or 5 days in the presence or absence of 10 μm hesperetin (DMSO as the vehicle). Cells surrounded with dotted red lines in the middle panels are magnified in the right panels. The cell surrounded by a white dotted line is a typically differentiated one with widespread membranes. (**B**) Differentiated cells are statistically depicted (** *p* < 0.01; *n* = 10 fields). (**C**) The lysates of cells at 5 days following the induction of differentiation were immunoblotted with the respective antibodies against differentiation markers PLP1 and MBP, cell lineage marker Sox10, and internal control actin. (**D**) Quantification of immunoreactive bands, using hesperetin plus immunoreactive bands as 100%, is depicted in the respective graphs of PLP1, MBP, Sox10, and actin (** *p* < 0.01; *n* = 3 blots).

**Figure 6:**
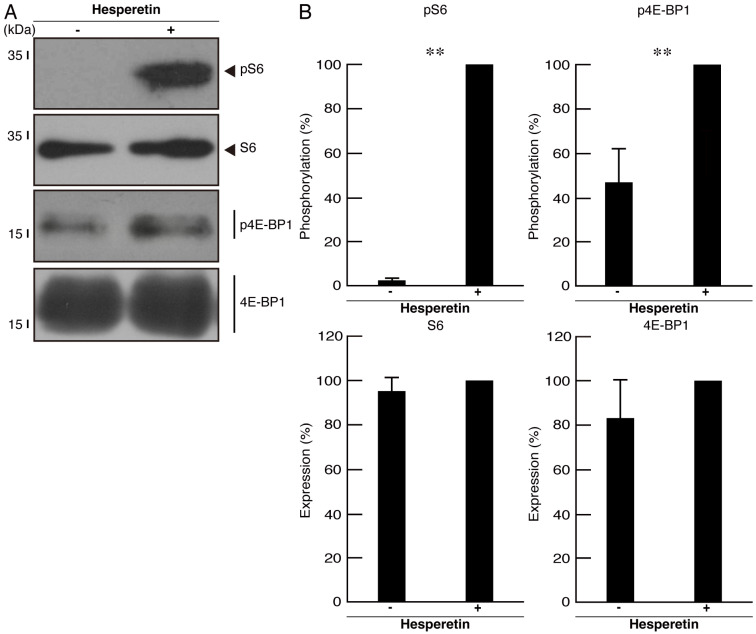
Hesperetin recovers decreased phosphorylation levels of ribosomal S6 and translational 4E-BP1 proteins in cells harboring mutated TMEM106B. (**A**) The lysates of cells at 5 days following the induction of differentiation in the presence or absence of hesperetin were immunoblotted with the respective antibodies against phosphorylated ribosomal S6 and translational 4E-BP1 proteins (pS6 and p4E-BP1). Total ribosomal S6 and translational 4E-BP1 protein (S6 and 4E-BP1) bands are also presented. (**B**) Quantification of immunoreactive bands, using hesperetin plus immunoreactive bands as 100%, is depicted in the respective graphs of pS6, S6, p4E-BP1, and 4E-BP1 (** *p* < 0.01; *n* = 3 blots).

We investigated whether hesperetin, a citrus flavonoid known as an mTOR signaling activator with cell-protective effects [25,26], recovered the phenotypes in cells harboring mutated TMEM106B. When we treated the cells harboring mutated TMEM106B with hesperetin, the cellular phenotypes were recovered (Figure 5A,B). Consistently, the expression levels of oligodendrocyte differentiation/myelination marker proteins were recovered (Figure 5C,D). To confirm the effect of hesperetin on the differentiating morphologies, we stained cells with an antibody against MBP. Hesperetin resulted in increased differentiation phenotypes (Figure S2C,D). Similarly, the phosphorylation levels of the ribosomal S6 and translational 4E-BP1 proteins in cells harboring mutated TMEM106B were also recovered (Figure 6A,B).

In addition, following treatment with hesperetin, mutated TMEM106B was present as organelle-like punctate structures (Figure S3). As observed in the subcellular distribution of wild-type TMEM106B, treatment with hesperetin localized the mutant TMEM106B to the lysosome (Figure S4), indicating that hesperetin recovers the phenotypes observed in mutated TMEM106B.

As control experiments, we confirmed the effect of hesperetin on the phenotypes in cells harboring wild-type TMEM106B. Hesperetin did not affect the cellular phenotypes, as indicated by marker protein expression (Figure S5), nor did it influence the phosphorylation levels of the ribosomal S6 and translational 4E-BP1 proteins (Figure S6). These findings suggest that hesperetin may specifically affect cells harboring mutated TMEM106B.

## 4. Discussion

It is well known that lysosome dysfunction and/or dysfunctional signaling around the lysosome is broadly linked to neurodegeneration [33,34]. Mutations in lysosome gene products including the TMEM106B protein are associated with many types of glial and neurological diseases [1,2,3,4,5,6]. Recent studies on brain diseases illustrate the role of the TMEM106B protein in regulating many aspects of lysosomal function [1,2,3,4,5,6]. It is believed that this protein is indispensable for lysosomal function in glial and neuronal cells [1,2,3,4,5,6]. For example, in the *TMEM106B*-deficient Oli-neu oligodendroglial cell line, the protein expression levels of LAMP1 and cathepsins D and L are greatly decreased, revealing that the TMEM106B protein contributes to supporting the biomaterial degradation abilities in the lysosomes in oligodendroglial cells [35]. In patients with genetic dementia, the expression levels in the neuronal and oligodendroglial cells of the brain tissue are downregulated; conversely, those of dementia-associated progranulin are upregulated [35]. It is likely that the decreased expression or deficiency of *TMEM106B* is linked to some brain diseases [5,6,36]. The TMEM106B protein is critically involved in controlling organelle function, such as the intracellular retrograde movement of lysosomes [1,2,3,4,5,6]. While the TMEM106B protein is a transmembrane protein in the lysosome [5,6], we have observed that HLD16-associated mutation causes the TMEM106B protein to affect Rab7-positive intracellular components. These components are known to correspond to late endosomes that link to the lysosomal organelle [31,32]. The HLD16-associated mutation may cause the TMEM106B protein to adopt an abnormal conformation, possibly forming small aggregates that are prone to degradation in the lysosome. This alteration could impair TMEM106B’s intended role on the cytoplasmic side of the lysosome.

There exists the possibility of a toxic gain of function for the HLD16-associated TMEM106B protein. This protein is also implicated in neurodegenerative diseases such as Alzheimer’s disease, Parkinson’s disease, amyotrophic lateral sclerosis (ALS), and frontotemporal lobar degeneration (FTLD) [37,38,39,40,41,42,43,44]. In addition, the BioGRID website (see https://thebiogrid.org/, accessed on 1 March 2024) bioinformatically identifies more than 300 molecules of actual and putative TMEM106B-binding proteins. Among those listed are some brain disease-associated proteins. Their protein complexes appear to be present under certain conditions, such as liquid–liquid phase separation (LLPS) in cytoplasmic and/or extracellular regions [45,46]. In FTLD-related risk single-nucleotide polymorphism (SNP) carriers, as one of the examples, mutated TMEM106B protein-derived filaments are increased, precisely correlating with the dysfunction of ALS type 10 (ALS10)-associated 43 kDa nuclear RNA/DNA-binding protein (TDP-43) [44]. It is possible that the differentially localized HLD16-associated TMEM106B mutant protein could undergo denaturation and aggregation, especially in long-term cell culture. Alternatively, the differentially localized TMEM106B mutant protein might inhibit the function of TMEM106B-binding proteins or other proteins. In either scenario, future studies will help to elucidate the cellular pathological changes occurring in myelinating oligodendroglial cells, which typically have a long lifespan.

It is unclear how the HLD16-associated mutation of *TMEM106B* decreases the phosphorylation levels of the ribosomal S6 and translational 4E-BP1 proteins as the output molecules of mTOR signaling. These proteins are typically localized around the lysosome as part of a molecular complex resembling a signalosome [47,48,49,50]. The TMEM106B protein has multifunctional roles in the lysosome, contributing to the maintenance of lysosomal homeostasis [5,6]. Therefore, mutated TMEM106B, which fails to be localized in the lysosome, may affect mTOR signaling around the lysosome to decrease the activity leading to the phosphorylation of the ribosomal S6 and translational 4E-BP1 proteins. However, it remains to be established whether the function and localization of TMEM106B are indeed linked to mTOR signaling [47,48,49,50].

It is noteworthy that signaling through mTOR plays an essential role in oligodendroglial cell differentiation and myelination [15,16]. Transgenic mice overexpressing Akt kinase, a central molecule in the mTOR signaling pathway, typically experience enhanced oligodendroglial cell differentiation and myelination, causing enhanced myelination [51]. Similarly, almost all positive regulators composed of mTOR signaling around the lysosome are involved in triggering proper myelination [52,53,54]. These findings suggest a possible connection between TMEM106B and mTOR signaling, which is required for proper myelination [2].

The naturally prenylated flavonoid fraction from *Glycyrrhiza glabra* or astilbin, as the major flavanonol derived from *Hypericum perforatum*, has the ability to stimulate the enzymatic activity of Akt kinase as well as mitogen-activated protein kinase (MAPK) [55,56]. Although it is unlikely that hesperetin is contained in the *G. glabra* or *H. perforatum* fraction, hesperetin or its unidentified hesperetin metabolic derivatives stimulate or modulate the enzymatic activity of Akt kinase, acting upstream or downstream of mTOR in some types of cells [57,58]. These effects may also be relevant in oligodendroglial cells [13,14,15,16]. It is thought that flavonoids including hesperetin reduce neuroinflammation, rather than directly affecting cells and tissue, in neurodegenerative diseases such as Alzheimer’s disease, Parkinson’s disease, and ALS [59,60]. However, although the direct intracellular molecular targets related to mTOR signaling are unknown so far, hesperetin has been reported to modulate the activity of several signaling molecules. One of them is tyrosine phosphatase 1B (PTP1B), which exhibits broad substrate specificity. PTP1B is a negative regulator of tyrosine phosphorylation for insulin receptor substrate 1 (IRS1) and molecules associated with mTOR signaling and Akt kinase, acting as direct downstream molecules of IRS1 [55]. Since these molecules are important for differentiation and myelination in oligodendroglial cells [15,16], it can be assumed that hesperetin directly promotes their differentiation and myelination by regulating the activity of PTP1B. While hesperetin target molecules such as PTP1B may not be therapeutic targets for underlying diseases such as HLD16, hesperetin can promote oligodendroglial cell differentiation and possible myelination in pathological conditions and reverse disease states, at least at the molecular and cellular levels.

In the present study, we observed that the HLD16-associated TMEM106B mutant protein is localized throughout the cytoplasmic region. Cells harboring mutated TMEM106B exhibit decreased morphological differentiation along with decreased ribosomal S6 and translational 4E-BP1 phosphorylation compared to those harboring the wild type. Conversely, hesperetin recovers the mutated TMEM106B-induced phenotypes. Further detailed studies will promote our understanding of not only the mechanism by which mutated TMEM106B decreases morphological differentiation in primary cells and mice but also the ways in which hesperetin can recover defective phenotypes. Such studies will allow us to clarify the molecular picture between the HLD16-associated TMEM106B mutant protein and signaling around the lysosome. Given that TMEM106B has been identified as the receptor required for the entry of SARS-CoV-2, independently of angiotensin-converting enzyme 2 (ACE2) [61,62], and it causes oligodendroglial pathological effects [63,64], studies focused on TMEM106B may reveal a link to oligodendroglial molecular and cellular pathologies that is stronger than previously anticipated.

## Figures and Tables

**Table 1:** Key materials used in this study.

Anti-lysosomal-associated membrane protein 1 (LAMP1)	Santa Cruz Biotechnology	sc-20011	J0919	IF, 1:100
Anti-Rab5	Santa Cruz Biotechnology	sc-46692	B2124	Immunoprecipitation (IP), 0.5 μg per 500 μg of cell extracts; immunoblotting (IB), 1:50
Anti-Rab7	Santa Cruz Biotechnology	sc-376362	F1023	IP, 0.5 μg per 500 μg of cell extracts; IB, 1:50
Anti-Arf6	Santa Cruz Biotechnology	sc-7971	E0919	IP, 0.5 μg per 500 μg of cell extracts; IB, 1:50
Anti-green fluorescent protein (GFP)	MBL	598	084	IF, 1:100,000; IB, 1:1000
Anti-myelin proteolipid protein 1 (PLP1)	Atlas Antibodies	HPA004128	8115828	IB, 1:1000
Anti-myelin basic protein (MBP)	BioLegend	836506	B225469	IB, 1:500; IF, 1:100
Anti-Sox10	Santa Cruz Biotechnology	sc-365692	J0720	IB, 1:500
Anti-actin (also called pan-bata-type actin)	MBL	M177-3	007	IB, 1:500
Anti-eIF4EBP1 (phosphorylated T37-specific)	abcam	ab75767	GR88680-14	IB, 1:2500
Anti-elF4EBP1	abcam	ab32024	GR239794-12	IB, 1:5000
Anti-ribosomal protein S6 (phosphorylated S240 and S244)	abcam	ab215214	GR3205097-3	IB, 1:10,000
Anti-ribosomal protein S6	Santa Cruz Biotechnology	sc-74459	D2921	IB, 1:500
Anti-IgG (H+L chain) (mouse) pAb-HRP	MBL	330	366	IB, 1:5000
Anti-IgG (H+L chain) (rabbit) pAb-HRP	MBL	458	354	IB, 1:5000
Alexa Fluor TM 488 goat anti-mouse IgG (H+L)	Thermo Fisher Scientific	A11001	774904	IF, 1:500
Alexa Fluor TM 594 goat anti-mouse IgG (H+L)	Thermo Fisher Scientific	A11005	2179228	IF, 1:500
Alexa Fluor TM 488 goat anti-rabbit IgG (H+L)	Thermo Fisher Scientific	A11008	751094	IF, 1:500
Alexa Fluor TM 594 goat anti-rabbit IgG (H+L)	Thermo Fisher Scientific	A11012	2018240	IF, 1:500
Hesperetin	Santa Cruz Biotechnology	sc-202647	D1921	Final concentration, 10 μm
Dimethyl sulfoxide (DMSO)	FUJIFILM Wako Pure Chemical Corporation	047-29353	CDN0170	Final concentration, less than 0.1%
pEGFP-C1-human TMEM106B	Synthesized by GeneScript	n.d.	n.d.	1.25 μg of DNA per 3.5 cm dish or 6 cm dish
pEGFP-C1-human TMEM106B (D252N)	Synthesized by GeneScript	n.d.	n.d.	1.25 μg of DNA per 3.5 cm dish or 6 cm dish
pEGFP-C1 (for mock transfection)	Isolated from pEGFP-C1-TMEM106B	n.d.	n.d.	1.25 μg of DNA per 3.5 cm dish or 6 cm dish

## Data Availability

The datasets used and/or analyzed in the current study are available from the corresponding author upon reasonable request.

## Supplementary Materials

The following supporting information can be downloaded at: https://www.mdpi.com/article/10.3390/cimb46080478/s1, Figure S1: Immunoprecipitation of respective antibodies against intracellular component marker proteins; Figure S2: Cells harboring mutated TMEM106B show decreased cell differentiating abilities; Figure S3: Following the treatment with hesperetin, TMEM106B mutant protein localizes to punctate structures; Figure S4: Following the treatment with hesperetin, TMEM106B mutant protein localizes to the lysosome; Figure S5: The effects of hesperetin on cells harboring wild type TMEM106B. Figure S6: The effects of hesperetin on phosphorylation levels of ribosomal S6 and translational 4E-BP1 proteins; Figure S7: Original-size images of immunoblots from figures; Figure S8: Original-size images of immunoblots from Supplemental Figure S1; Figure S9: Original-size images of immunoblots from Supplemental Figures S5 and S6.
