# Supplementary material for: Hypomyelination Leukodystrophy 16 (HLD16)-Associated Mutation p.Asp252Asn of TMEM106B Blunts Cell Morphological Differentiation
Source: Curr Issues Mol Biol. 2024 Jul 27;46(8):8088–103. doi: 10.3390/cimb46080478 (PMC11352280; doi:10.3390/cimb46080478)
Supplement: Supplementary file 1 [file cimb-46-00478-s001.zip › 02.2. Supporting .pdf]

Supplemental figure legends

Figure S1. Immunoprecipitation of respective antibodies against intracellular component marker proteins. Cells transfected with the plasmid encoding EGFP-tagged wild type protein or its mutant were extracted using an isotonic extraction buffer. Immunoprecipitation was performed with the respective antibodies against intracellular components Arf6 (A), Rab5 (B), and Rab7 (C), followed by immunoblotting with anti-GFP and the respective antibodies against intracellular components. The extracts were also immunoblotted with an anti-GFP antibody.

Figure S2. Cells harboring mutated TMEM106B show decreased cell differentiating abilities. (A, B) Cells harboring wild type (WT) or mutated (D252N) TMEM106B were allowed to differentiate for 0 or 5 days. They were stained with an anti-MBP antibody, and cells with two or more secondary processes were statistically expressed as a percentage (** $p < 0.01$; n = 10 fields). (C, D) Cells harboring mutated (D252N) TMEM106B were allowed to differentiate for 0 or 5 days in the presence of vehicle or hesperetin (Hes). They were stained with an anti-MBP antibody, and cells with two or more secondary processes were statistically expressed as a percentage (** $p < 0.01$; n = 10 fields).

Figure S3. Following the treatment with hesperetin, TMEM106B mutant protein localizes to punctate structures. (A) FBD-102b cells (surrounded by white dotted lines) were transfected with the plasmid encoding EGFP-tagged mutant protein and treated with

hesperetin. Transfected cells were stained with DAPI to detect nuclear positions. Scan plots were performed along the white dotted lines in the direction of the arrows in the images. (B) Graphs showing the green and blue fluorescence intensities (F. I., arbitrary units) along the white dotted lines in the direction of the arrows were presented in the bottoms of representative fluorescence images. (C) Cells with abnormal, widely distributed structures were counted and statistically depicted (** $p < 0.01$; n = 10 fields).

Figure S4. Following the treatment with hesperetin, TMEM106B mutant protein localizes to the lysosome. (A) FBD-102b cells (surrounded by white dotted lines) were transfected with the plasmid encoding EGFP-tagged mutant protein and treated with hesperetin. Transfected cells were stained with the respective antibodies against KDEL, GM130, and LAMP1. Scan plots were performed along the white dotted lines in the direction of the arrows in the images. (B) Graphs showing the green and red fluorescence intensities (F. I., arbitrary units) along the white dotted lines in the direction of the arrows were presented in the bottoms of representative fluorescence images. (C) The respective merged percentages were depicted in bar graphs (n = 3 fields).

Figure S5. The effects of hesperetin on cells harboring wild type TMEM106B. (A) Cells harboring wild type TMEM106B were allowed to differentiate for 0 or 5 days in the presence or absence (vehicle control only) of hesperetin. Cells surrounded by dotted red lines in the middle panels were magnified in the right panels. The cell surrounded by a white dotted line indicates a typically differentiated cell with widespread membranes. (B)

Differentiated cells were statistically depicted (n = 10 fields). (C) Cell lysates at 5 days post-differentiation induction were immunoblotted with the respective antibodies against differentiation markers PLP1 and MBP, cell lineage marker Sox10, and the internal control actin. (D) Quantification of immunoreactive bands, using control immunoreactive bands as 100%, was depicted in the respective graphs for PLP1, MBP, Sox10, and actin (n = 3 blots).

Figure S6. The effects of hesperetin on phosphorylation levels of ribosomal S6 and translational 4E-BP1 proteins. (A) Cell lysates at 5 days post-differentiation induction were immunoblotted with the respective antibodies against phosphorylated ribosomal S6 and translational 4E-BP1 proteins (pS6 and p4E-BP1). Total ribosomal S6 and translational 4E-BP1 protein (S6 and 4E-BP1) bands were also presented. (B) Quantification of immunoreactive bands, using control immunoreactive bands as 100%, was depicted in the respective graphs of pS6, S6, p4E-BP1, and 4E-BP1 (n = 3 blots).

Figure S7. Original-size images of immunoblots from figures.

Figure S8. Original-size images of immunoblots from supplemental figure 1.

Figure S9. Original-size images of immunoblots from supplemental figures 5 and 6.
