# Supplementary figures and images for: Hypomyelination Leukodystrophy 16 (HLD16)-Associated Mutation p.Asp252Asn of TMEM106B Blunts Cell Morphological Differentiation
Source: Curr Issues Mol Biol. 2024 Jul 27;46(8):8088–103. doi: 10.3390/cimb46080478 (PMC11352280; doi:10.3390/cimb46080478)

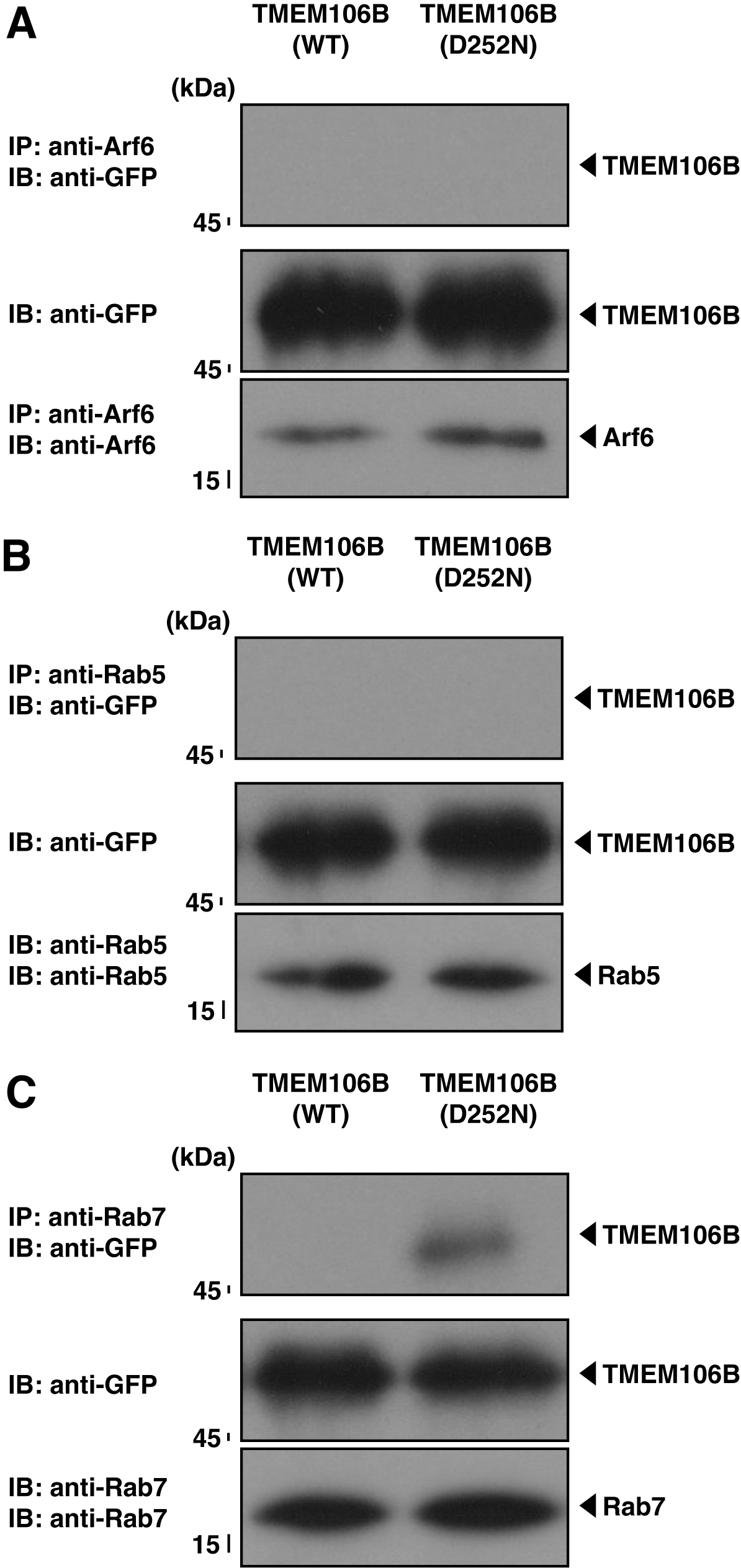

Supplement: Supplementary file 1 [file cimb-46-00478-s001.zip › Figure S1.tif]

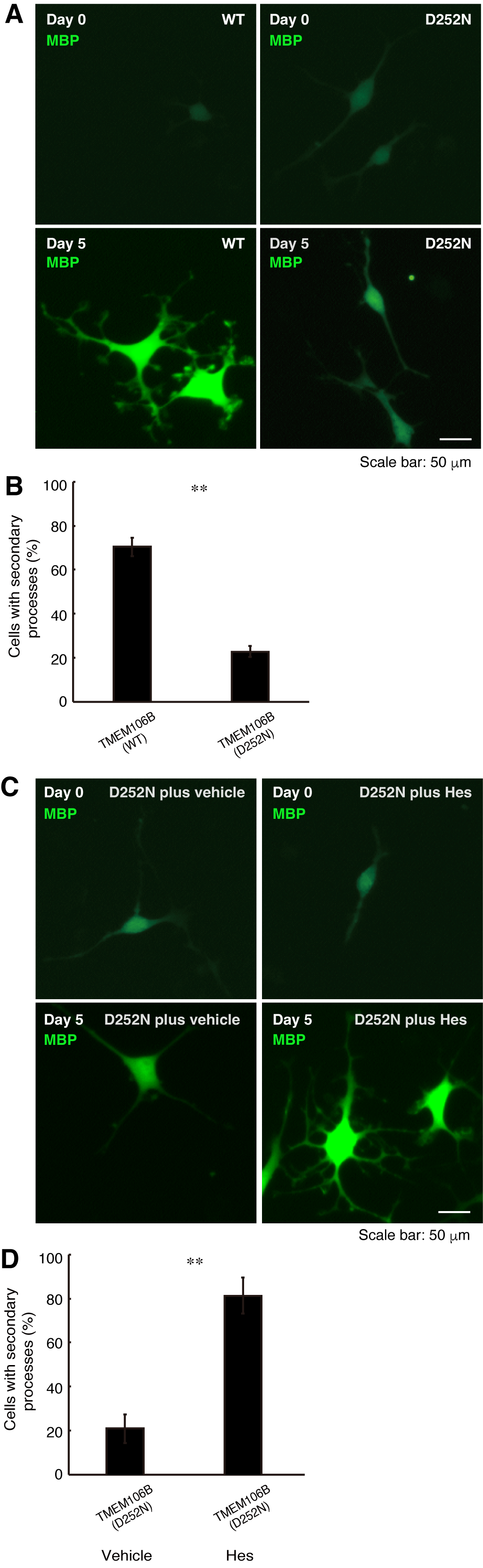

Supplement: Supplementary file 1 [file cimb-46-00478-s001.zip › Figure S2.tif]

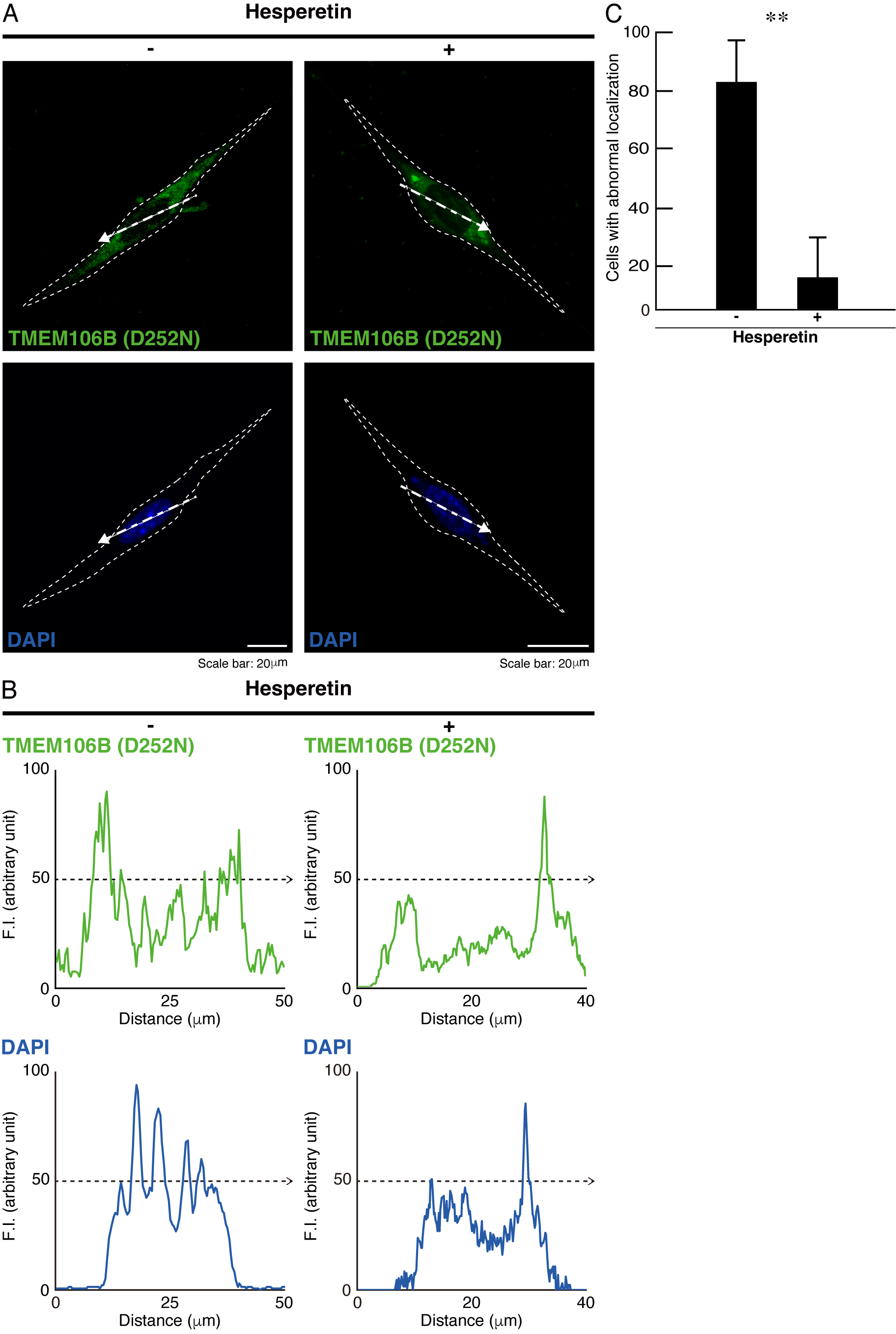

Supplement: Supplementary file 1 [file cimb-46-00478-s001.zip › Figure S3.tif]

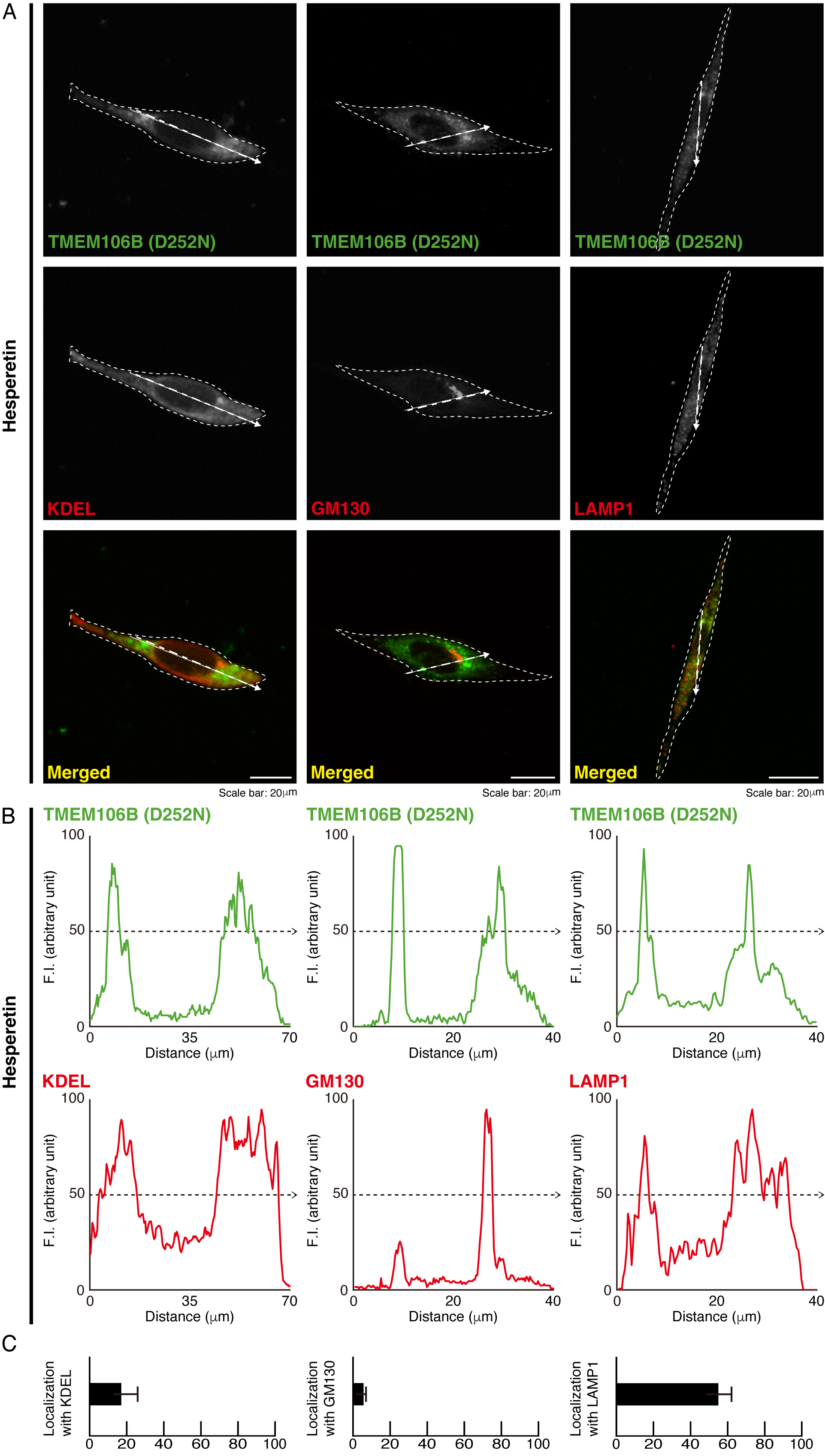

Supplement: Supplementary file 1 [file cimb-46-00478-s001.zip › Figure S4.tif]

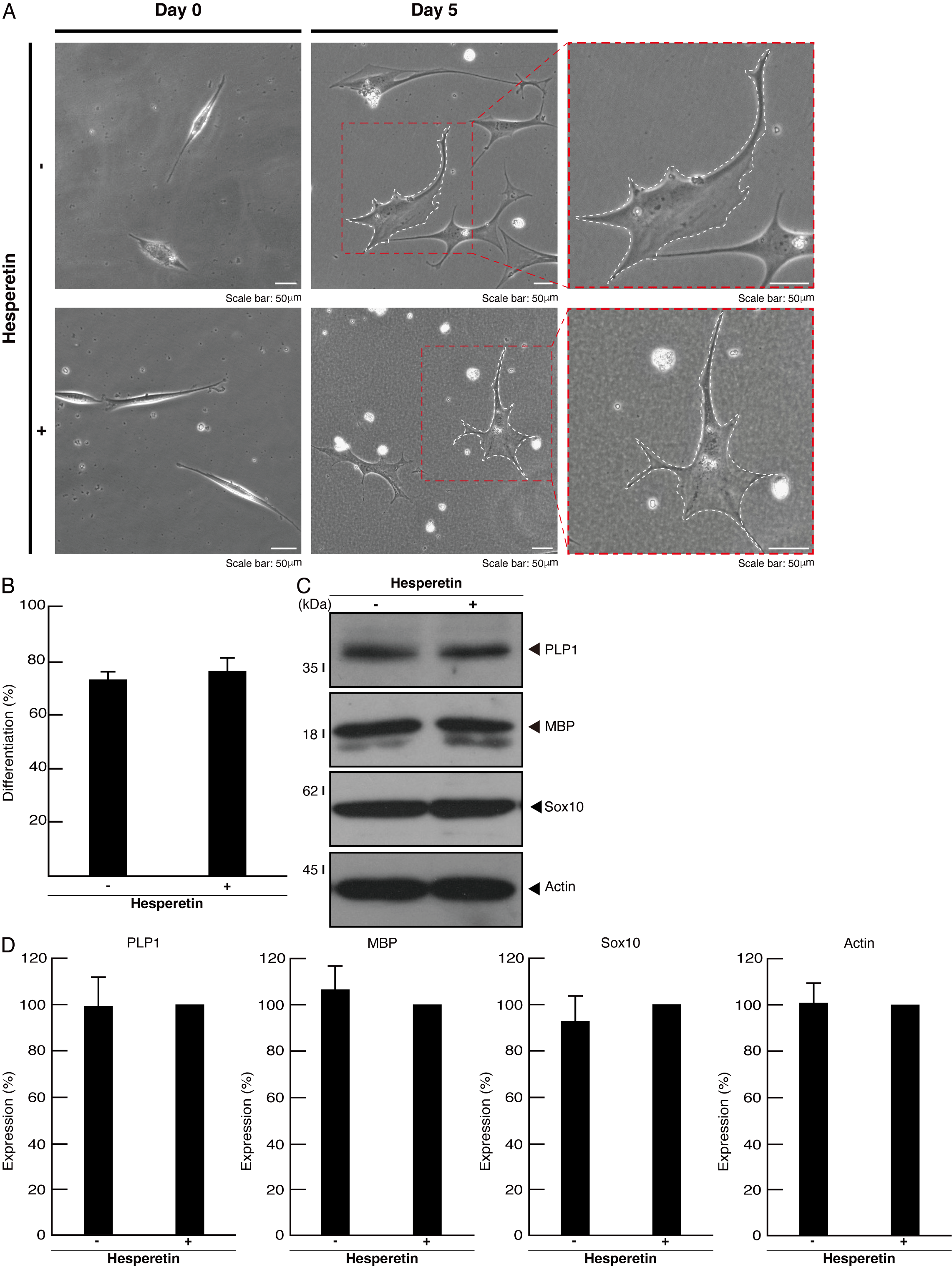

Supplement: Supplementary file 1 [file cimb-46-00478-s001.zip › Figure S5.tif]

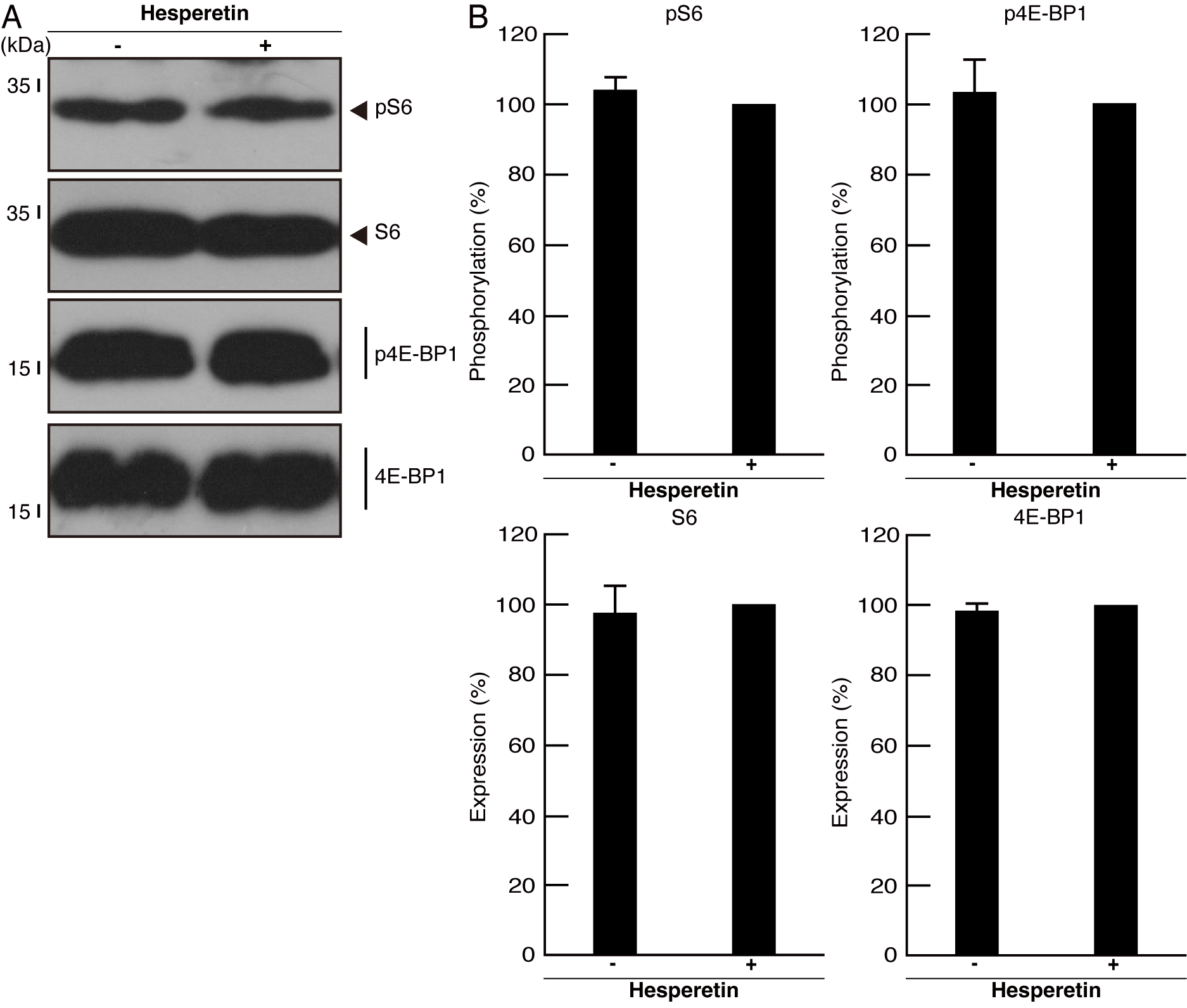

Supplement: Supplementary file 1 [file cimb-46-00478-s001.zip › Figure S6.tif]

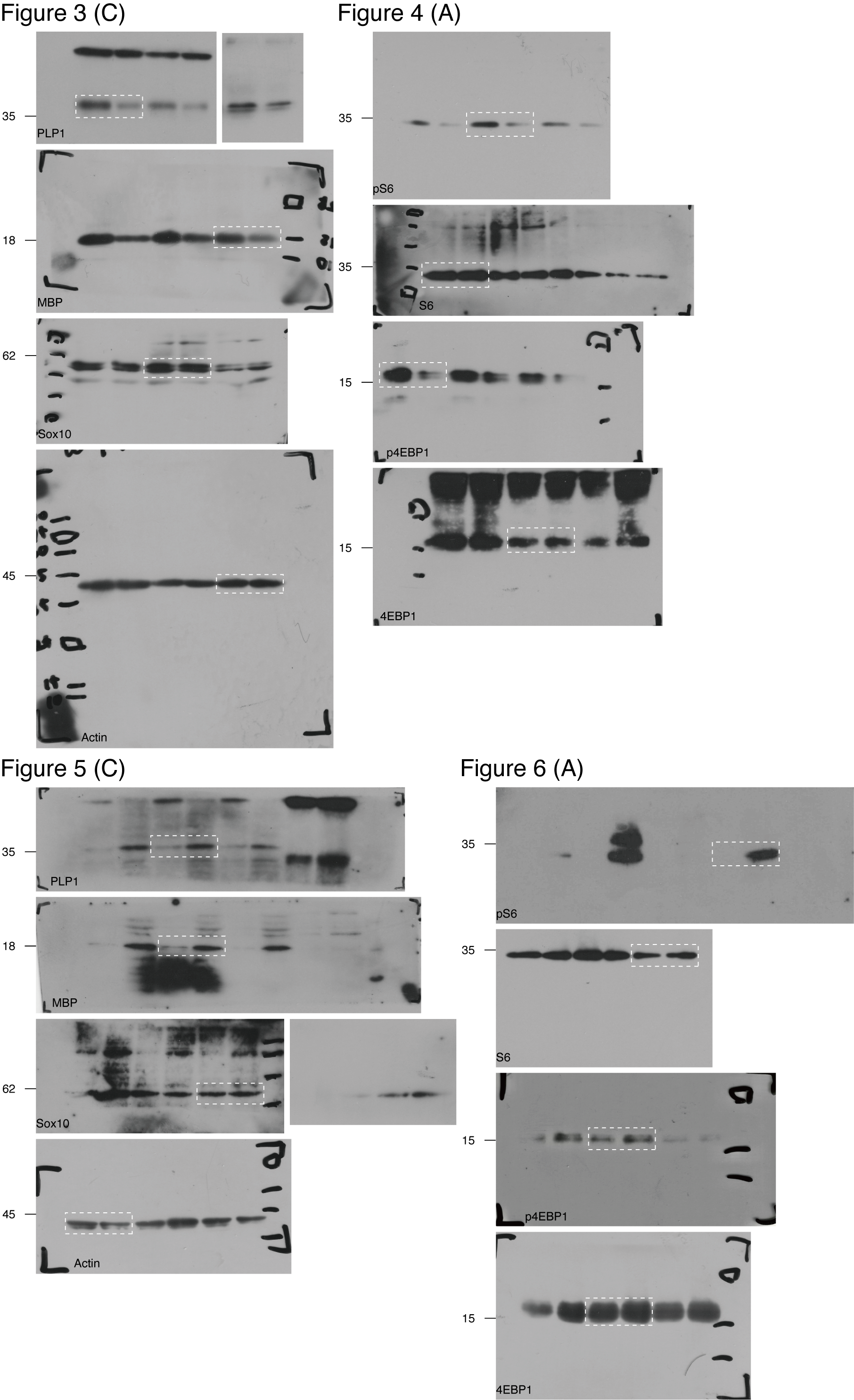

Supplement: Supplementary file 1 [file cimb-46-00478-s001.zip › Figure S7.tif]

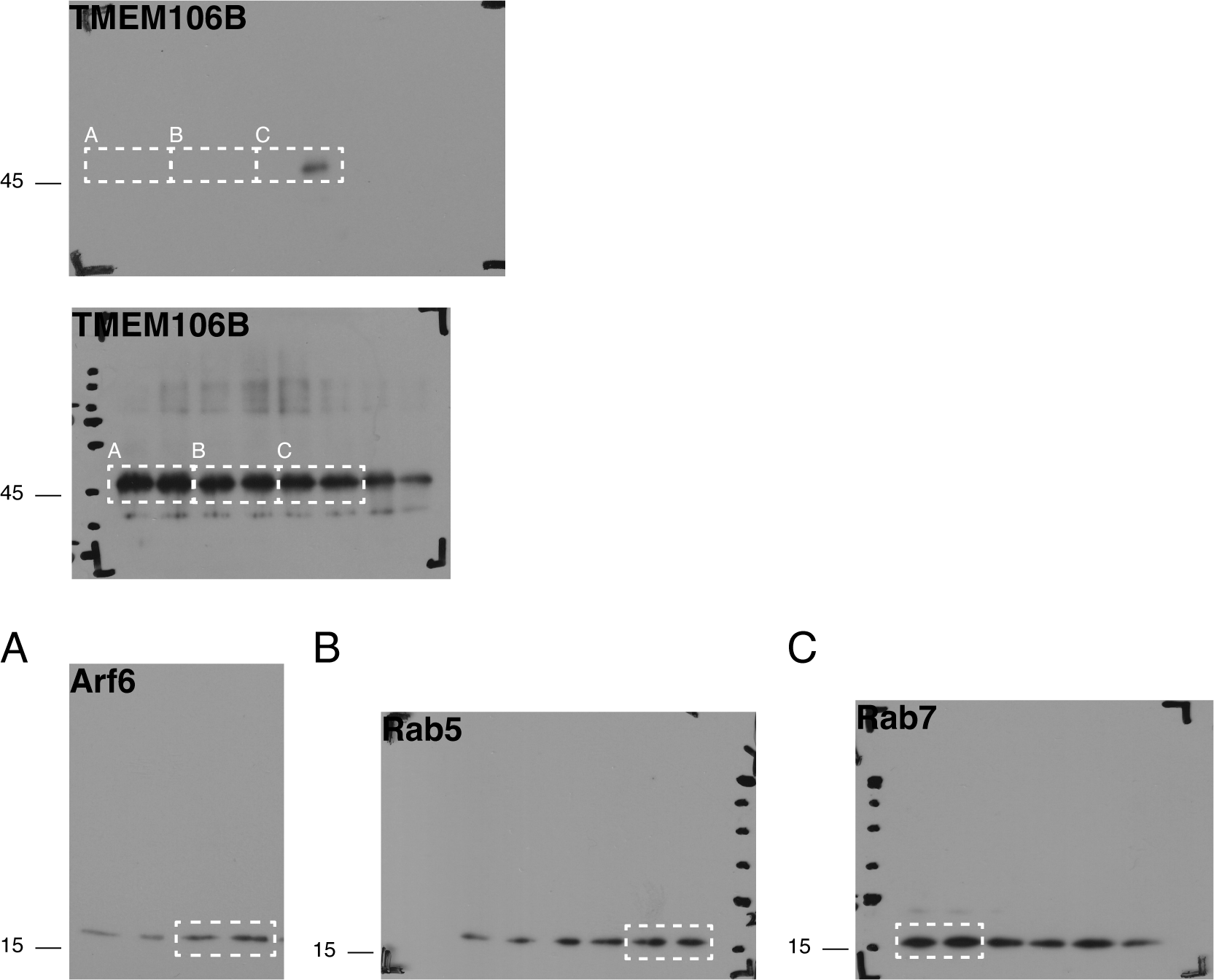

Supplement: Supplementary file 1 [file cimb-46-00478-s001.zip › Figure S8.tif]

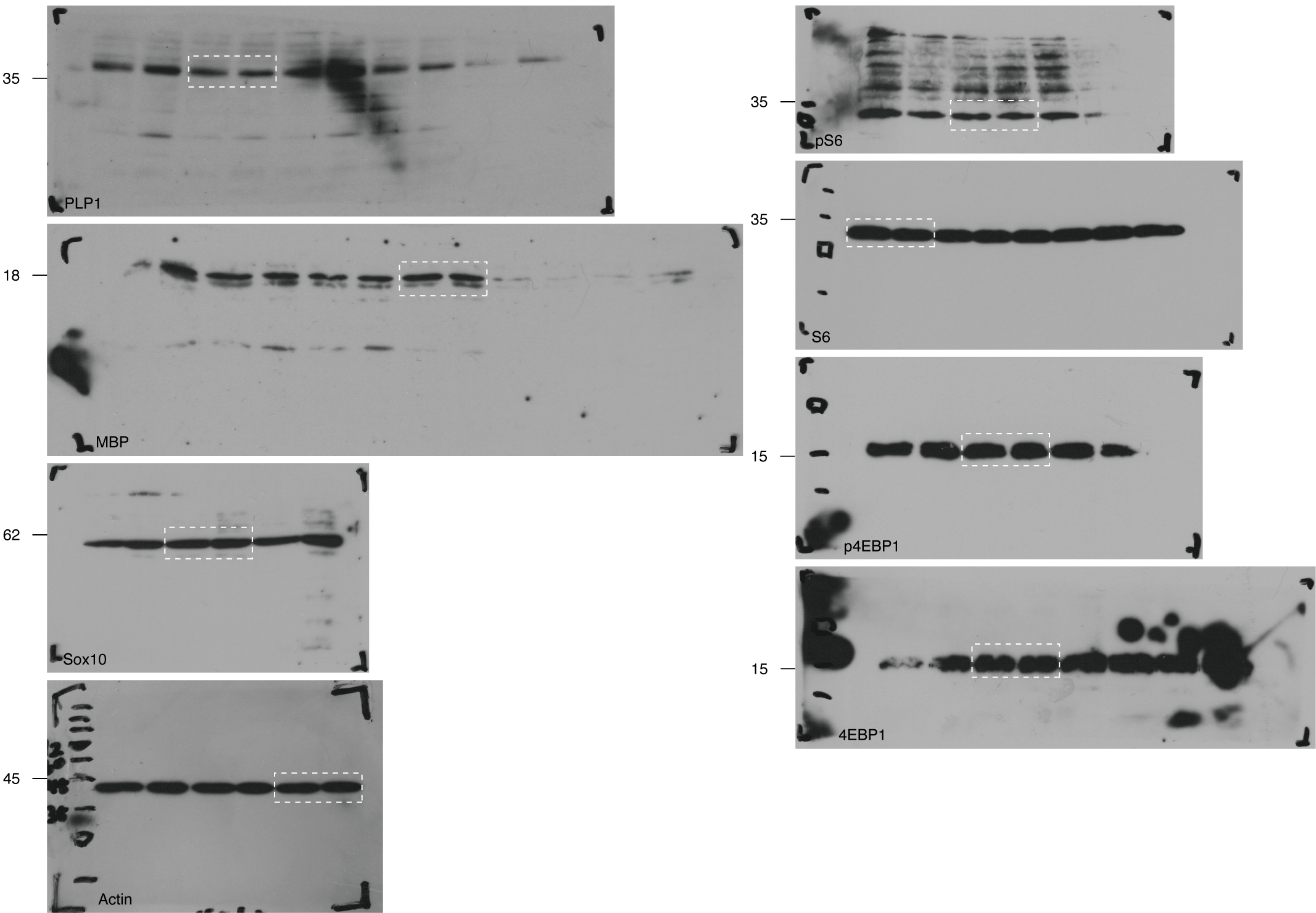

Supplement: Supplementary file 1 [file cimb-46-00478-s001.zip › Figure S9.tif]
